# Food-related nanoemulsions: Fabrication approaches, characterization methods, and emerging applications

**DOI:** 10.1016/j.crfs.2026.101591

**Published:** 2026-10-10

**Authors:** Zhi-Hong Zhang, Kaiwei Zhang, Xiaolan Li, Yuyue Qin, Jingkun Yan, Xianli Gao

**Affiliations:** a School of Food Science and Engineering, Jiangsu University, Zhenjiang, 212013, China; b School of Pharmacy, Jiangsu University, Zhenjiang, 212013, China; c Faculty of Food Science and Engineering, Kunming University of Science and Technology, Kunming, 650500, China; d School of Life and Health Technology, Dongguan University of Technology, Dongguan, 523808, China

**Keywords:** Food-grade nanoemulsion, Bioactive delivery, Food quality, Edible packaging, Functional food

## Abstract

Nanoemulsions are thermodynamically metastable but kinetically durable colloidal carriers with droplet sizes typically below 500 nm, capable of encapsulating lipophilic bioactive compounds to enhance their solubility, storage stability and *in vivo* bioavailability. Their small droplet size provides a large specific surface area, high encapsulation efficiency, and improved oral bioavailability, thereby alleviating the poor water solubility and oxidative degradation of polyphenols, essential oils, and fat-soluble compounds. This review provides an overview of two mainstream fabrication routes of food-grade nanoemulsions, namely high-energy and low-energy emulsification, and compares their operating principles, production costs, industrial scalability, and inherent limitations. Common characterization techniques for particle size, surface charge, microstructure, rheological properties, and biological activity are discussed, with a comparative analysis of their respective detection advantages and applicable scenarios. Recent research advances over the past five years (alongside foundational references for technical background) in food-related applications are summarized, covering three core directions: food quality preservation, biodegradable active packaging, and nutrient-fortified functional foods. Critical research gaps in current studies are highlighted, including insufficient systematic *in vivo* safety data and limited industrial-scale validation of natural pigment delivery systems, particularly regarding long-term stability during processing and storage. Furthermore, targeted future research priorities centered on green low-cost fabrication, all-natural emulsifier development, and stimulus-responsive delivery system construction are proposed. This review aims to provide theoretical insights and technical guidance for accelerating the translation of nanoemulsion platforms toward sustainable health-promoting food applications.

## Highlights

• High- and low-energy fabrication strategies for food-grade nanoemulsions are compared.
• Food preservation, active packaging, and functional food applications are summarized.
• Evidence gaps in safety, scale-up, and *in vivo* validation are identified.
• A formulation-to-industrial-translation evidence framework is proposed.
• Nanoemulsions show broad application prospects in health-related fields.

## Graphical abstract

**Figure:**
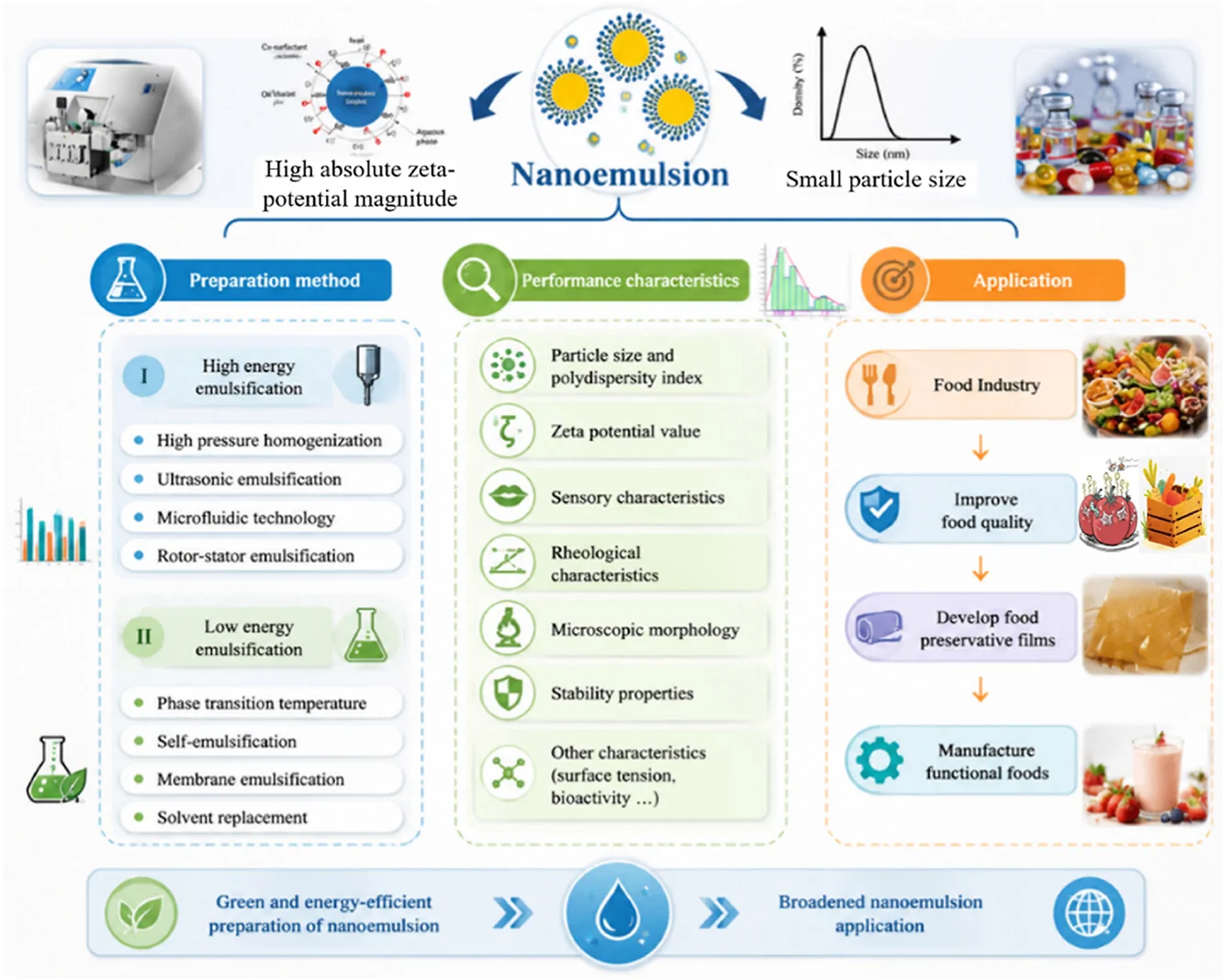

## 1 Introduction

Growing consumer demand for clean-label and nutrient-enhanced foods has driven the development of efficient delivery vehicles for natural functional ingredients (Aguilera, 2025; Hossain et al., 2025; Pan et al., 2025; Shishir et al., 2024). Polyphenols, plant essential oils, lipophilic vitamins, and natural pigments possess excellent antioxidant and antibacterial properties, but their practical applications in foods are severely restricted by low water solubility, rapid thermal and photodegradation, and low intestinal absorption efficiency (Mahajan et al., 2026; C. Shen et al., 2022; Waqar et al., 2026; Z.-H. Zhang et al., 2024). Traditional micron-scale emulsions are prone to aggregation and phase separation during long-term storage (Gandhi et al., 2026; Qu et al., 2025). In contrast, nanoemulsions with droplet sizes typically below 500 nm maintain favorable long-term colloidal stability primarily through strong Brownian motion that counteracts gravitational separation, along with electrostatic repulsion that depends on interfacial charge and solution conditions. This improved stability significantly enhances the solubility and stability of hydrophobic bioactive compounds, as well as their bioavailability due to the micellization efficiency driven by lipid composition (Cheng et al., 2026; He et al., 2023; Jin et al., 2026; Zhang et al., 2025, Zhang et al., 2025, Zhang et al., 2025, Zhang et al., 2025). In this review, “food-grade nanoemulsion” is defined as a colloidal dispersion stabilized by a food-grade emulsifier, in which all ingredients—including the oil phase, emulsifier, cosurfactant, solvent, polymer and encapsulated bioactive substances—meet the regulatory purity standards and are used for oral administration within the established acceptable daily intake limit. Beyond bioactive delivery, growing concerns over conventional plastic food packaging have also encouraged the development of nanoemulsion-enabled biodegradable and intelligent packaging systems.

Bibliometric analysis based on Web of Science records spanning 2016 to 2026 retrieved 7909 publications concerning nanoemulsions. To ensure a systematic and reproducible review, the search was restricted to English-language articles and reviews using keywords including “food-grade nanoemulsion”, “fabrication”, “characterization”, and “application”. After duplicate removal and screening according to the inclusion criteria (food-grade nanoemulsion, focusing on manufacturing or application), eligible articles were selected for analysis. Food science constitutes the second-largest research discipline with 1567 relevant papers, and annual publication output maintains a steady upward trend; partial data for 2026 are presented as the database has not yet completed full annual indexing (Fig. 1A and B). The continuous growth of publications from 2016 to 2026 reflects persistent academic attention and broad development potential of nanoemulsion carriers within food research. The VOSviewer keyword co-occurrence network was generated based on publications from 2020 to 2026, capturing the most recent research hotspots in this field. Moreover, the VOSviewer-generated keyword co-occurrence network (Fig. 1C) further partitions the field into four color-coded clusters: blue for nanoemulsion fabrication and physicochemical stability, yellow-purple for essential oil nanoemulsions in food preservation and edible packaging, green for bioactive protection, biosafety and research prospects, and red for in vitro/*in vivo* evaluation and oral bioactive efficacy, with node size corresponding to keyword frequency and line thickness representing co-occurrence intensity. The VOSviewer analysis (version 1.6.20) used full counting, with a minimum keyword occurrence threshold of 10, association strength normalization, and keyword cleaning by merging synonyms and removing generic terms to ensure analytical robustness. However, bibliometric clustering suggests a certain degree of thematic segregation across research topics, as indicated by the four distinct clusters with limited inter-cluster connections (Fig. 1C). Several recent reviews have summarized nanoemulsion preparation, physicochemical properties, food applications, nutraceutical delivery, and related safety or scale-up issues (Kaur et al., 2024; Manzoor et al., 2023; Mushtaq et al., 2023). However, the present review differs by integrating fabrication, characterization, and food applications within a formulation-to-translation framework, with particular emphasis on comparative analysis, industrial scalability, evidence level, long-term stability, and *in vivo* safety. To address these gaps, the present review: (i) systematically compares high- and low-energy fabrication approaches with attention to industrial scalability; (ii) presents a multi-dimensional characterization framework linking colloidal properties to functional performance; (iii) surveys recent food applications (with emphasis on 2020–2026, alongside foundational references for classical methods) across quality preservation, active packaging, and functional foods; and (iv) identifies specific research priorities, including industrial-scale validation and long-term stability assessment of natural pigment-loaded nanoemulsions, as well as systematic *in vivo* safety evaluation, to guide future investigations.

**Fig. 1:**
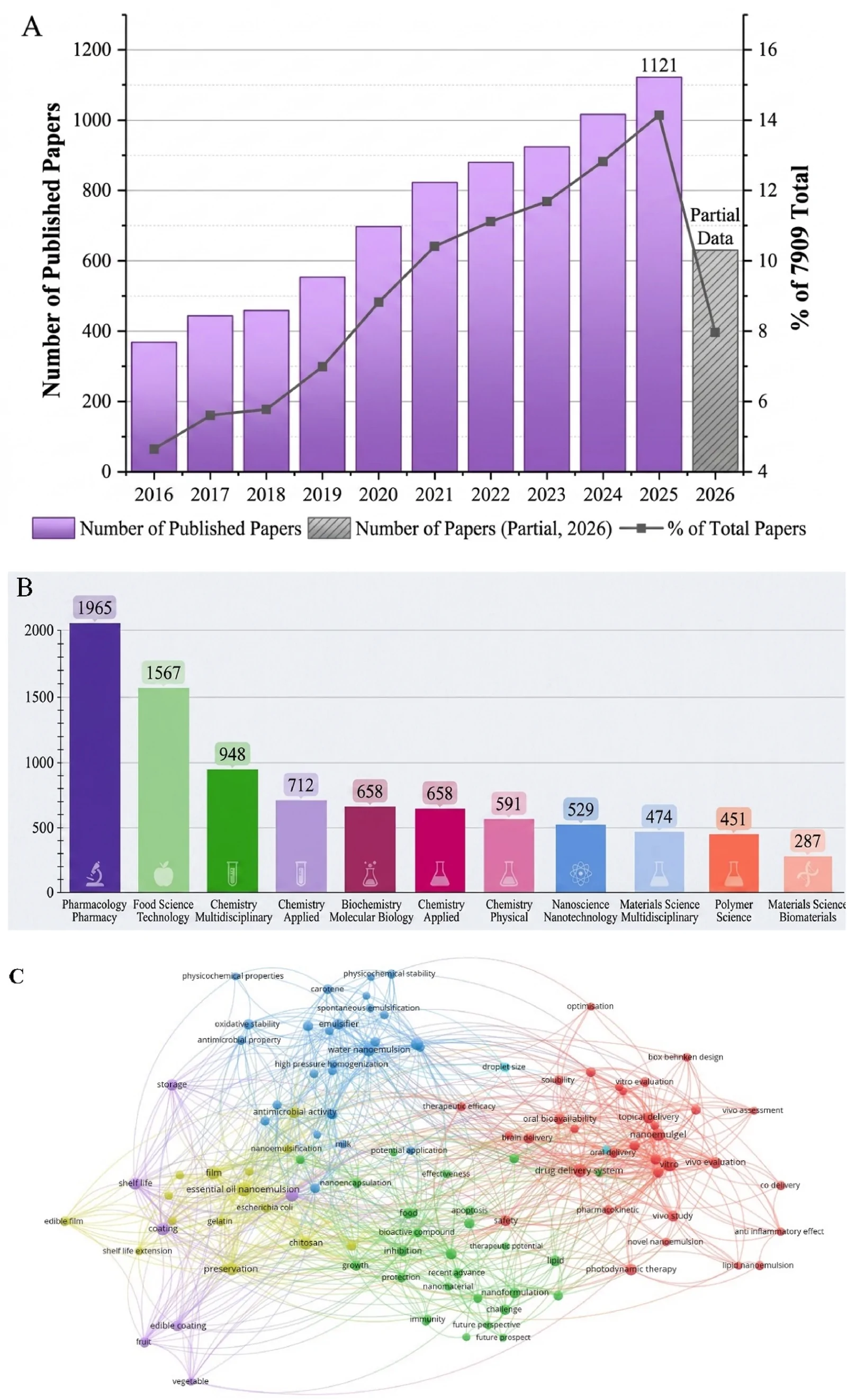
Bibliometric analysis of nanoemulsion research. (A) Annual publication trends from 2016 to 2026 based on Web of Science Core Collection (search date: July 15, 2026; document types: Articles and Reviews; language: English). Data for 2026 are partial due to incomplete annual indexing. (B) Distribution of the 7909 retrieved publications across research disciplines; Food Science accounts for 1567 papers (19.8%), representing the second-largest contributing field. (C) Keyword co-occurrence network generated by VOSviewer (version 1.6.20; full counting; minimum occurrence threshold = 10; association strength normalization; keyword cleaning by merging synonyms and removing generic terms) based on publications from 2020 to 2026.

## 2 Fabrication strategies of food-grade nanoemulsions

As kinetically stable non-equilibrium colloids, nanoemulsions cannot be formed spontaneously from oil and water alone, and thus require external energy input or physicochemical manipulation for fabrication. Based on energy intensity and assembly mechanism, preparation approaches are classified into high-energy emulsification and low-energy emulsification strategies (Al-Maqtari et al., 2021; Shishir et al., 2026). This classification reflects differences in energy input, physical principles, equipment requirements, and industrial scalability. As illustrated in Fig. 2A, nanoemulsion fabrication methods can be broadly categorized into high-energy and low-energy approaches, each encompassing several representative techniques based on distinct emulsification mechanisms. The mechanisms, merits, drawbacks, and applicable scenarios of these techniques are summarized in Table 1.

**Fig. 2:**
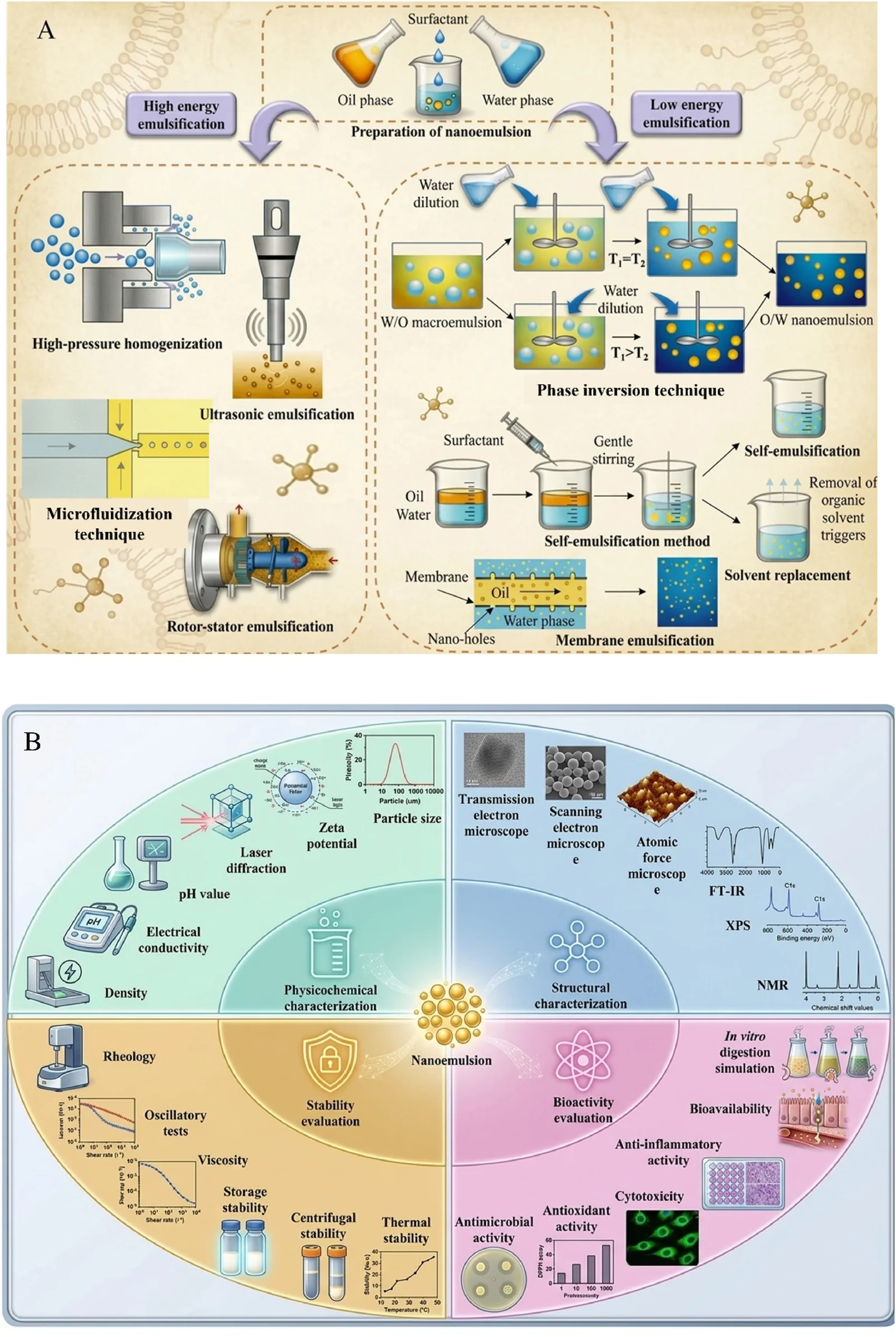
Technical roadmap of preparation (A) and characterization (B) of nanoemulsion.

**Table 1:** The mechanisms, merits, drawbacks, and applicable scenarios of nanoemulsion technologies.

Energy intensity	Preparation method	Mechanism of action	Major advantages	Major limitations	Scale/application suitability
High-energy emulsification method	High pressure homogenization method	Large droplets are dispersed into small droplets by using strong shearing force, strong impact force and other forces.	High throughput, controllable operating parameters, reproducible processing, and applicability to continuous production (Wang et al., 2019).	The emulsion system with reduced efficiency in highly viscous systems, high equipment cost and high energy consumption, potential degradation of heat-/shear-sensitive bioactives (Vladisavljević, 2016).	Large-scale industrial production
	Ultrasonic emulsification method	Ultrasonic vibration generates strong shear force and pressure gradient in emulsion, large droplets are refined.	Reduces energy loss and emulsifier requirements and optimizes the amount of emulsifier, with simple operation, low cost and low-energy consumption (Bhavya et al., 2021; Thokchom et al., 2020).	Generally, recycling is needed; Risk of localized overheating, which may affect the stability of heat-sensitive active ingredients (Yukuyama et al., 2016).	Small-scale laboratory research
	Microfluidization technology	High-speed flowing phases generated under high pressure collide with each other.	It has high controllability and accuracy, balanced stress on the sample, relatively low mechanical damage to sensitive components and a small amount of surfactant, and can continuously produce nanoemulsions (García-Toledo et al., 2019; Gong et al., 2019).	Equipment and maintenance costs are high (Ozturk and Turasan, 2022; Yukuyama et al., 2016).	Small-scale production
	Rotor-stator emulsification method	Using the mechanical force and hydrodynamic effect between rotor and stator, liquid materials are subjected to strong shear, friction and impact.	Easy installation, low-cost investment and viscous system compatibility (Burapapadh et al., 2016; Scholz and Keck, 2015).	The prepared emulsion has a large droplet size and needs further homogenization (Roselan et al., 2020).	Medium-scale industrial production
Low-energy emulsification method	Phase inversion temperature (PIT) method	Triggers phase inversion by temperature-induced changes in nonionic surfactant hydration	Simple implementation and low cost	Generally restricted to suitable nonionic surfactant systems, and high concentration of surfactants is needed (Espitia et al., 2019)	Medium-scale production
	Phase inversion composition (PIC) method	Triggers phase inversion by progressively changing the oil/water ratio at constant temperature	Low-energy requirement, simple operation, and no need for expensive equipment (Feng et al., 2020).	High concentration of surfactant is needed, and the requirements for component ratio and mixing conditions are high.	Medium-scale production
	Self-emulsification method	Using the self-assembly characteristics of amphiphilic molecules, a uniformly dispersed emulsion system is spontaneously formed driven by interfacial-energy minimization/spontaneous interfacial processes.	Optimize the energy production rate, potential for scale-up, improve the loading capacity, and the operation is simple and insensitive to temperature changes (Li et al., 2020).	Requires strict control of formulation ratios, and its application scope is limited by specific oil-water system (Cholakova et al., 2022).	Large-scale industrial production
	Membrane emulsification method	The dispersed phase is pressed into the continuous phase through the microporous membrane, and the emulsion is prepared by using the pore size and interfacial tension of the membrane.	Simple operation, mild operating conditions, low-energy consumption and mild processing with limited damage to sensitive components, dispersed phase and continuous phase.	The equipment and maintenance costs are high, and the requirements for membrane aperture and operating parameters are strict, and the application scope is limited (Sneha & Kumar, 2022).	Small-scale production
	Solvent replacement method	After the organic phase is mixed with the water phase, the organic solvent is quickly removed, and the self-assembly of nano-droplets is induced by the change of interfacial energy.	Simple operation and wide application range, and is especially suitable for encapsulating hydrophobic drugs or active ingredients.	The removal of organic solvents increases the complexity of the process, and requires higher selection and dosage of solvents (Azmi et al., 2019).	Laboratory research

### 2.1 High-energy emulsification method

High-energy methods employ intense mechanical forces generated by specialized equipment to disrupt the dispersed phase into nanoscale droplets, enabling uniform dispersion within the continuous phase and forming stable colloidal systems. These processes rely on shear stress, impact forces, and cavitation to repeatedly fragment and restructure droplets, facilitating ordered alignment of emulsifiers at the oil–water interface and enhancing system stability. Widely used high-energy techniques include high-pressure homogenization, ultrasonic emulsification, microfluidization, and rotor–stator homogenization (Karim et al., 2024; Mushtaq et al., 2023).

#### 2.1.1 High-pressure homogenization

High-pressure homogenization is a scalable industrial technique for producing nanoemulsions (<200 nm). Crude emulsions flow through narrow valves under ultrahigh pressure, and synergistic shear, impact and cavitation break large oil droplets into uniform nanoscale dispersions (Choi and McClements, 2023). Homogenization pressure, cycle number and oil–water viscosity ratio dominate droplet size evolution (L. J. Li et al., 2023). Standard industrial settings cover 50–100 MPa (max. 350 MPa), 2–5 cycles, and 20–35 °C constant cooling; low temperature offsets friction/cavitation heat to protect thermolabile bioactives. Within a rational range, higher pressure and repeated cycles shrink droplet size, yet over-homogenization causes droplet re-aggregation and destabilization (Zhang et al., 2025). Shi et al. (2022) prepared 125 nm pepper essential oil nanoemulsions with Tween 80; three homogenization cycles at 50 MPa strengthened the emulsion's centrifugal, pH and thermal tolerance as well as its antibacterial activity. Ge et al. (2024) fabricated chitosan-tremella polysaccharide stabilized astaxanthin nanoemulsions via three homogenization cycles at 83 MPa, with improved pigment photostability and oral bioavailability.

This method features high throughput, controllable parameters, stable batch performance and wide food matrix compatibility, supporting medium-scale continuous production. Its major drawbacks include costly equipment and high energy input. Intense shear and instantaneous temperature surges trigger protein denaturation, polysaccharide depolymerization and lipid oxidation, damaging heat/shear-sensitive functional components (Heidary and Soltanizadeh, 2024; Mushtaq et al., 2023). Additionally, highly viscous feedstocks hinder energy transfer, drastically lowering droplet fragmentation efficiency (Signori-Iamin et al., 2025).

#### 2.1.2 Ultrasonic emulsification method

Ultrasonic emulsification relies on high-frequency sound waves to generate cavitation. Violent collapse of cavitation bubbles generates strong shear and shockwaves, breaking the dispersed oil phase into nanoscale droplets (Barbosa-Cánovas et al., 2022; Li et al., 2021, Li et al., 2021; Shah et al., 2024). Droplet size declines with elevated ultrasonic power, amplitude and treatment duration under appropriate ranges, with the optimized minimal droplet size falling within 50–150 nm (Amiri-Rigi et al., 2023). Zhang et al. (2022) prepared sodium caseinate-stabilized seabuckthorn oil nanoemulsions with a minimal droplet size of 238.2 nm at 450 W for 20 min. Gholamhosseinpour et al. (2023) fabricated uniform *Satureja sahendica* essential oil nanoemulsions (473.2 nm, PDI = 0.182) under 200 W, 226 kHz ultrasonication; the system maintained good physical stability for 14 days and exhibited strengthened antioxidant and antibacterial activity against foodborne pathogens. Sousa et al. (2026) adopted pre-emulsification coupled with pulsed high-intensity sonication (40% amplitude, 5 min) to produce cholinium oleate-stabilized β-carotene nanoemulsions. Higher sunflower oil dosage improved emulsion storage stability and the in vitro gastrointestinal bioaccessibility of encapsulated β-carotene.

This technique features simple operation, low equipment cost and moderate energy input, supporting fine tuning of droplet size and efficient emulsifier utilization (Qayum et al., 2023; Thokchom et al., 2020). Nevertheless, uneven acoustic energy distribution requires repeated treatment to reach uniform droplet distribution. High-viscosity feedstocks weaken emulsification efficiency, and localized overheating causes degradation of heat-labile bioactives (Juliato et al., 2025). Therefore, these drawbacks restrict the scalable industrial application of ultrasonic emulsification.

#### 2.1.3 Microfluidization technology

Microfluidization pushes crude emulsions through microscale reaction chambers via ultrahigh-pressure pumps. Supersonic jet collision, hydraulic shear and localized cavitation jointly break large droplets into homogeneous nanodispersions. This top-down treatment drives ordered emulsifier arrangement at oil–water interfaces and generates nanoemulsions with a narrow size distribution and steady colloidal stability (Zhang et al., 2025). Unlike conventional high-pressure homogenization, microfluidization employs fixed-geometry interaction chambers where pressurized fluid streams collide at high velocity, generating droplet breakup through a combination of supersonic jet collision, high shear, and intense cavitation under a more uniform flow field.

Zhang et al. (2021) prepared a microfluidized pea protein–curcumin dispersion (357.45 nm, zeta potential 33.43 mV) by microfluidization, which maintained good storage and thermal stability. Its manufacturing principle and colloidal behavior provided a useful reference for studies of nanoemulsions. Combined with transglutaminase crosslinking, microfluidization further strengthened interfacial molecular assembly and physical stability of whey protein-stabilized nanoemulsions (Chen et al., 2023).

Standard operational parameters cover 50–207 MPa pressure, 1–5 cycles, and temperature below 40 °C to avoid thermal degradation of sensitive substances; optimized formulations produce droplets ranging 50–150 nm (Kim et al., 2019). Microfluidization delivers precise size control, narrow particle distribution and low surfactant dosage, while mild mechanical force limits bioactive loss, fitting lab-scale and small-batch precise preparation (Gong et al., 2019). Restrictive drawbacks include expensive hardware and maintenance, frequent microchannel blockage, and poor scalability for mass industrial manufacturing (Ozturk and Turasan, 2022).

#### 2.1.4 Rotor-stator emulsification

Rotor–stator homogenization is a cost-effective mechanical emulsification technique that relies on high-speed rotational motion within a confined stator gap to generate strong fluid shear and turbulent disturbance. Such hydrodynamic forces disrupt the oil–water interface and break coarse bulk phases into fine dispersed droplets (Gazolu-Rusanova et al., 2020). This high-shear equipment excels at fast bulk mixing and is well matched to thick, high-viscosity raw materials, often adopted as a pre-processing emulsification unit. Heyn et al. (2021) reported that the semi-flexible whey protein amyloid fibrils decreased in length from 6200 nm to a minimum of 190 nm under rotor-stator shearing. This reduction was comparable to that achieved by high-pressure homogenization (283–111 nm). Niknam et al. (2022) optimized rotor–stator parameters (20,000 rpm, 10.1 min) to prepare water-in-oil nanoemulsions loaded with phenolic-rich olive cake extract, achieving a droplet size of 105.8 nm and PDI of 0.255; after 30 days of storage, 90.2% antioxidant activity and 83.1% polyphenol content were retained.

Standard operating ranges cover rotating speeds of 10,000–25,000 rpm, treatment durations between 5 and 15 min, and fixed rotor-stator gaps of 0.2–0.5 mm. Under regular single-pass treatment, the achievable droplet dimension falls within 100–500 nm (Gazolu-Rusanova et al., 2020; Matman et al., 2022). This homogenizer type brings obvious economic merits including low installation expense and broad tolerance to viscous food substrates, fitting mid-volume pre-emulsification production lines. However, single-stage rotor-stator shearing generally does not generate droplets that meet standard nanoemulsion specifications, and supplementary high-pressure homogenization or ultrasonication is required to narrow particle size distribution for improved physical stability and nutrient digestibility (Dueñas-López et al., 2026; Sneha & Kumar, 2022).

### 2.2 Low-energy emulsification method

Low-energy emulsification relies on spontaneous molecular interactions inside oil–water mixtures rather than strong mechanical input to assemble nanoscale dispersions (Li et al., 2020). Uniform tiny droplets form automatically by adjusting formulation composition and environmental conditions to reduce overall interfacial energy and trigger controllable phase shifts. Compared with high-energy fabrication routes, this mild processing mode cuts energy expenditure and protects thermally or mechanically fragile bioactive substances from structural damage. The formation and long-term stability of resultant nanoemulsions are jointly regulated by multiple factors: surfactant category and concentration, oil-phase physicochemical traits, co-surfactant or co-solvent addition, together with external ionic strength and temperature fluctuations (da Silva, do Rosario, Weitz and Conte-Junior, 2022). Four widely adopted low-energy emulsification techniques for food nanoemulsions are discussed in subsequent sections.

#### 2.2.1 Phase inversion technique

##### 2.2.1.1 Phase inversion temperature (PIT) method

The PIT method triggers temperature-dependent phase inversion by modulating the hydration state and hydrophilic–lipophilic balance (HLB) of nonionic surfactants (e.g., ethoxylated Tweens and Spans). At low temperatures, surfactants are hydrophilic and stabilize O/W emulsions; as temperature rises to the PIT, dehydration shifts affinity toward lipophilicity, forming bicontinuous microemulsion intermediates with ultra-low interfacial tension; rapid cooling then locks in nanoscale droplets as kinetically stable nanoemulsions, distinguishing the final nanoemulsion from the transient microemulsion state (Aswathanarayan and Vittal, 2019). Liu et al. (2025) optimized a cyclohexane/Span 80–Tween 80/water system at 65 °C, achieving optimal stability with viscosity < 2.5 mPa s and uniform sub-200 nm droplets. P. H. Chen et al. (2025) prepared ginger essential oil nanoemulsions with a particle size of 145.6 nm and zeta potential of −42.4 mV using a low-temperature continuous phase transition method derived from the PIT principle, which effectively inhibited microbial growth, retarded lipid oxidation and pH elevation in prepared duck meat, and prolonged the shelf life by approximately 21 days.

Typical parameters involve a preparation temperature of 50–80 °C (10–15 °C above PIT), cooling rate > 10 °C/min, and surfactant concentration of 10–20 wt%; the minimum droplet size is 100–200 nm (Espitia et al., 2019). Merits include low-energy input, simple operation, and no expensive equipment; limitations include strict restriction to nonionic surfactants, high surfactant demand, and narrow formulation compatibility, suitable for medium-scale production of nonionic surfactant-based systems.

##### 2.2.1.2 Phase inversion composition (PIC) method

The PIC method induces phase inversion at a constant temperature by systematically modulating the relative proportions of oil, water, and surfactant, thereby altering the spontaneous curvature of the interfacial film. This process transitions from oil-surfactant mixtures to transient bicontinuous microemulsions and ultimately to stable oil-in-water (O/W) nanoemulsions via kinetic trapping through controlled aqueous phase dilution, driven primarily by compositional variations rather than thermal effects (Jiang et al., 2020). Zhang et al. (2020) developed DHA/EPA-loaded nanoemulsions using a Tween 80/Span 80 (3:1) blend, achieving average sizes of 122.23 nm (DHA) and 112.73 nm (EPA) with narrow PDI (<0.17) and high encapsulation efficiency.

Typical parameters include surfactant-to-oil ratio of 4:6 to 7:3, stirring speed of 300–600 rpm, and stepwise aqueous phase addition; the minimum achievable size is 30–150 nm. Advantages include mild conditions, operational simplicity, low cost, and fine compositional tunability for complex systems (Yukuyama et al., 2016). Drawbacks include high emulsifier demand, strict ratio requirements, and process repeatability affected by component interactions, suitable for medium-scale formulation development (Feng et al., 2020).

#### 2.2.2 Self-emulsification method

Self-emulsification is a low-energy bottom-up strategy that spontaneously forms nanoemulsions via chemical potential gradients and interfacial self-assembly, without intensive mechanical input. When the oil-surfactant mixture contacts the aqueous phase, rapid solubilization and bicontinuous intermediate formation generate uniform nanodroplets driven by interfacial energy minimization (Espitia et al., 2019). Nazari et al. (2019) fabricated transparent sesame oil nanoemulsions (∼67 nm) via spontaneous emulsification, with size controlled by emulsifier type, phase ratio, and stirring speed. Panwar et al. (2024) confirmed that slow oil-phase feeding and optimized stirring (400–450 rpm) reduced droplet size and improved uniformity, enhancing industrial process feasibility. Shen et al. (2025) developed surfactant-free self-emulsifying systems for cyclosporine A delivery to improve oral bioavailability, which provided valuable mechanistic insights for developing food-grade nanoemulsions without emulsifiers.

Key parameters include surfactant concentration of 8–16 wt%, stirring speed of 300–600 rpm, feeding rate < 2 mL/min, and temperature insensitivity (25–60 °C); the minimum droplet size is 50–150 nm (Liew et al., 2020). Advantages include ultra-low energy consumption, excellent large-scale scalability, high bioactive loading, and thermal insensitivity, ideal for industrial mass production (Yang et al., 2023). Limitations include strict oil-surfactant compatibility, high surfactant demand, and sensitivity to formulation composition, restricting application to specific oil–water systems (Cholakova et al., 2022).

#### 2.2.3 Membrane emulsification method

Membrane emulsification extrudes the dispersed phase through uniform nanoporous membranes into the flowing continuous phase, generating monodisperse nanodroplets via controlled interfacial tension and hydrodynamic resistance. This gentle, size-tunable process minimizes mechanical stress and preserves bioactive integrity (Luo and Wei, 2023). Jiang and Charcosset (2022) combined pre-emulsification and membrane emulsification to prepare curcumin nanoemulsions with 94.5–96.5% encapsulation efficiency and precisely tunable droplet sizes via membrane pore modulation. Soussi et al. (2025) reported the development of water-in-oil-in-water (W_1_/O/W_2_) double emulsions via membrane emulsification for the co-encapsulation of catechin and L-lysine, achieving nanoscale internal droplets (149.40 nm) and excellent thermal stability during pasteurization, and the formulated double emulsions effectively improved the microbial safety, antioxidant properties, and storage quality of carrot-based infant purées. In addition, Mondal et al. (2024) fabricated monodisperse hydrophobic deep-eutectic-solvent-based nanoemulsions (average size = 147 nm, PDI = 0.22) via a sustainable membrane emulsification technique using a reusable laser-drilled stainless-steel isoporous membrane, demonstrating excellent antibacterial and antifungal activities.

Typical parameters include transmembrane pressure of 10–60 bar, membrane pore size of 30–150 nm, continuous phase flow rate of 10–50 mL/min, and room-temperature operation; the minimum droplet size is 50–150 nm (Karabulut et al., 2025). Merits include mild conditions, low-energy input, exceptional monodispersity, and minimal bioactive damage, ideal for lab-scale precision formulation. Limitations include high membrane costs, fouling/clogging risks, low throughput, and poor scalability, restricted to small-scale and low-viscosity systems (Kaade et al., 2023).

#### 2.2.4 Solvent replacement method

Solvent displacement dissolves lipophilic bioactives in water-miscible organic solvents (ethanol, acetone), which diffuse into the aqueous surfactant phase under gentle stirring; solvent evaporation then induces self-assembly of uniform nanodroplets via interfacial energy shifts. It should be noted that solvent displacement can yield nanoemulsions, nanocapsules, or nanoparticles depending on the oil phase state and polymer-to-oil ratio, requiring case-by-case structural verification. This method enables high encapsulation of poorly soluble actives under mild conditions (Walia et al., 2019). Cerda-Opazo et al. (2021) prepared procyanidin-enriched nanoemulsions with ∼160 nm size, PDI ≤ 0.1, zeta potential ∼−50 mV, and ∼97% encapsulation efficiency, stable across salinity, pH, and temperature gradients. Heidarifard et al. (2021) developed curcumin-loaded coconut oil nanoemulsions using the solvent displacement method, achieving controllable particle sizes and high encapsulation efficiency, which were subsequently incorporated into pH-responsive alginate hydrogels with excellent antibacterial activity and sustained release behavior.

Key parameters include organic solvent-to-water ratio of 1:1 to 1:3, stirring speed of 500–1000 rpm, evaporation temperature of 30–45 °C, and surfactant concentration of 5–10 wt%; the minimum droplet size is 50–200 nm (Katre et al., 2021). Advantages include simple operation, broad compatibility with hydrophobic actives, and fine size control, suitable for lab-scale research and high-value formulation development (Azmi et al., 2019). Drawbacks include organic solvent residues requiring removal, increased process complexity, high solvent consumption, and environmental and safety concerns. For food applications, residual solvents must be reduced to regulatory limits through rigorous post-processing steps such as rotary evaporation or vacuum drying, which further limits the industrial scalability of this method for food-grade nanoemulsions (Heidarifard et al., 2021).

## 3 Performance characteristics of nanoemulsions

Clarifying the composition, microstructural features and inherent physicochemical traits of food nanoemulsions lays a solid foundation for their targeted design, performance tuning and quality evaluation. Droplet features, interfacial film structure, colloidal stability and environmental responsiveness jointly dominate the functional behavior of nanoemulsions, including encapsulation capacity, delivery efficiency and storage stability within complicated food systems, as displayed in Fig. 2B. Full characterization of the above indicators helps compare various emulsification routes, screen favorable manufacturing conditions and optimize processing parameters to obtain high-efficiency delivery systems. Multiple analytical tools are adopted to quantify droplet morphology, interfacial properties, colloidal stability and environmental response of nanoemulsions. Moreover, key characterization indicators and analytical methodologies of representative nanoemulsions are systematically compared in Table 2.

**Table 2:** Key characterization indicators and analytical methodologies of representative nanoemulsions.

Nanoemulsion	Formulation	Preparation method	Particle size (nm)	Zeta potential (mV)	Microscopic morphology	Stability	Other characteristics	References
β-carotene	Complex of corn protein hydrolysis (CPH) and tannic acid (TA)	Ultrasonic homogenization	155.5	−40∼-50	Laser scanning confocal microscopy (LSCM)	The nanoemulsion exhibited good physical and chemical stability during the 30-day storage period, but the digestive stability had no obvious effect		Wang et al. (2024)
	Olive oil, beta-carotene and soybean lecithin	Ultrasonic homogenization	112.36		LSCM	The nanoemulsion exhibited good stability under varying ionic strength, pH, freeze–thaw, storage, and oxidative conditions	Variation of turbidity and total chromatic aberration	Mehmood et al. (2021)
	Ternary conjugate of soybean protein isolate (SPI), (EGCG) and maltose	Ultrasonic homogenization	139.6	−32.9	LSCM	It has good storage, light and thermal stability		Geng et al. (2023)
Curcumin	MCT,TW80,Lecithin and QCS	Ultrasonic homogenization	218.71	−27.6	TEM	It has good storage, ionic, thermal and pH stability	FT-IR; antibacterial and antioxidant activity analysis	Zhu et al. (2023)
	Olive oil and castor oil, natural polysaccharide	Ultrasonic homogenization	35.77	−29.34	TEM		Rheology, thermodynamics, FT-IR and antioxidant capacity analysis	Richa and Choudhury (2020)
	Tween 20	Ultrasonic homogenization	138.1	−20.80∼-27.60	TEM	It has good stability under freeze-thaw conditions	Turbidity, antioxidant activity; in vitro digestion simulation	Sharma et al. (2021)
Vitamin E nanoemulsion	Sunflower seed oil, Span 80 and Tween 80	Solvent replacement method	118.37	−3.88	Environmental scanning electron microscopy (ESEM)		Encapsulation efficiency; in vitro release behavior	Harun et al. (2021)
	Soybean oil, soybean fat-soluble protein (LP)	High pressure homogenization method	100 ∼200	−5.0	AFM、LSCM、cryo-scanning electron microscopy (cryo-SEM)	Excellent emulsifying stability and antioxidant stability	Encapsulation efficiency; rheological characterization	Wang et al. (2021)
	Cold pressed linseed oil (FSO), octenyl succinic anhydride modified starch (OSA-MS) and Tween-80	Microfluidic technology	139			Excellent physical stability and antioxidant stability	Rheological characteristics; in vitro digestion simulation; high performance liquid chromatography bioaccessibility	Maldonado et al. (2023)
*Thymus vulgaris* Oil nanoemulsion	Essential oils of clove and thyme, Tween-80	Ultrasonic homogenization	68.6		TEM		Antibacterial and anti-biofilm activities	Hashem et al. (2024)
	Thyme essential oil, Tween-80	Ultrasonic homogenization	91.28		TEM		Antibacterial activity	Doghish et al. (2023)
	Thyme essential oil (Th-EO), cellulose nanocrystals (CNC) and Tween-80	Solvent replacement method	230	−25∼-27	SEM、TEM	The storage stability and centrifugal stability are good within two weeks	Determination of total phenol content; Antibacterial performance analysis	Baldassarre et al. (2023)

### 3.1 Droplet size distribution and polydispersity index (PDI)

Droplet size distribution and polydispersity index (PDI) represent the most fundamental structural parameters governing colloidal stability, bioaccessibility, and functional performance of nanoemulsions (Aswathanarayan and Vittal, 2019). Dynamic light scattering (DLS) and laser diffraction are the most widely employed analytical techniques for quantitative characterization. DLS enables accurate measurement of nano-to-submicron particles by analyzing fluctuations in scattered light intensity induced by Brownian motion, whereas laser diffraction determines particle size based on angle-dependent light diffraction patterns, with diffraction angle inversely proportional to particle diameter (Kim et al., 2023; Mushtaq et al., 2023). Notably, electron microscopy coupled with image analysis has emerged as a powerful orthogonal method for visualizing and quantifying droplet morphology and size distribution with nanometer resolution. Optimal nanoemulsions for food applications typically exhibit droplet diameters below 200 nm, which generate an ultrahigh specific surface area, enhance Brownian motion, and strengthen electrostatic repulsion, thereby effectively inhibiting creaming or sedimentation. For instance, Saari et al. (2020) reported that curcumin-loaded nanoemulsions with an average diameter of 19.92 nm and PDI of 0.17 demonstrated exceptional stability and bioavailability owing to highly uniform size distribution. Similarly, perilla oil nanoemulsions optimized at 120 MPa homogenization pressure achieved a minimum droplet size of 293.87 ± 6.55 nm, exhibiting enhanced oxidation stability and oral absorption efficiency (Hu et al., 2021). Droplet size is synergistically modulated by formulation composition (surfactant-to-oil ratio, oil phase viscosity) and processing parameters (homogenization pressure, cycle number, energy input), providing a versatile strategy for tailoring nanoemulsion performance (Valdivia-Olivares et al., 2023).

PDI is a dimensionless parameter derived from the cumulants analysis of dynamic light scattering (DLS) data, representing the variance of the intensity-weighted distribution of diffusion coefficients. Values below 0.2 are often considered indicative of a relatively narrow size distribution. Decreased droplet size and PDI collectively enhance resistance to physical instability, whereas aggregation and coalescence phenomena broaden size distribution and increase PDI, compromising system stability (Kampa et al., 2022). Zhou et al. (2021) developed mannitol monooleate-stabilized nanoemulsions with diameters ranging from 47.51 to 599.46 nm and PDI values of 0.33–0.42, demonstrating controllable size modulation through formulation engineering. Collectively, precise control of droplet size distribution and PDI represents a foundational strategy for optimizing encapsulation efficiency, controlled release kinetics, and colloidal stability of food-grade nanoemulsions (Delforce et al., 2023).

### 3.2 Zeta potential

Zeta potential quantifies the electrostatic potential at the slipping plane of colloidal droplets, serving as a definitive indicator of interdroplet electrostatic repulsion and long-term colloidal stability (Tran et al., 2021). Sufficient surface charge generates strong electrostatic repulsion forces that counteract van der Waals attraction, effectively suppressing droplet aggregation and coalescence (Ahmadi and Jafarizadeh-Malmiri, 2020). Conventionally, absolute zeta potential values exceeding 30 mV indicate good stability, whereas values below 20 mV typically signal insufficient electrostatic stabilization and heightened instability risk (Xue and Luo, 2023). However, these thresholds are not universal, particularly for sterically stabilized systems containing nonionic surfactants, proteins, or polysaccharides, where stability can be maintained even at low zeta potentials through steric hindrance. Therefore, zeta potential should be interpreted in conjunction with other stability indicators, including interfacial composition, ionic strength, pH, and rheological behavior.

Zeta potential is predominantly governed by emulsifier type, pH, ionic strength, and interfacial composition. Anionic nanoemulsions stabilized by proteins or polysaccharides typically exhibit negative zeta potentials, while cationic systems can be engineered using chitosan or peptide surfactants (Rai et al., 2018). For example, linseed oil nanoemulsions with a zeta potential of −41.0 mV demonstrated exceptional storage stability, while tea seed oil nanoemulsions with zeta potential of −12.33 mV exhibited rapid phase separation in their respective studies (Mazonde et al., 2020). Although these two formulations differ in oil composition and processing conditions, the zeta potential may have played a role in the observed stability difference. Notably, zeta-potential measurements in concentrated systems require rigorous dilution protocols to avoid multiple scattering effects, and phase-specific analysis can provide insights into interfacial charge distribution (Fürst et al., 2023). Recent advances have demonstrated that zeta potential modulation through interfacial engineering represents a powerful strategy for enhancing stability under food processing conditions (thermal, pH, and ionic stress) and controlling gastrointestinal digestion kinetics (Wei et al., 2021).

### 3.3 Sensory characteristics

Visual inspection provides a rapid and direct means of assessing the macroscopic uniformity, phase separation, sedimentation, and particle aggregation of nanoemulsion formulations. These critical indicators are essential for evaluating both physical stability and overall product quality. Phase separation can lead to emulsion delamination, compromising uniformity; precipitation indicates that some components may aggregate or settle; and particle aggregation alters the particle size distribution, affecting both stability and functional characteristics. The presence of these abnormalities often signals instability issues within the formulation, which can adversely impact performance in practical applications (Fu et al., 2022). Thus, visual observation plays a vital role in quality control, ensuring that the nanoemulsion meets industry standards and aligns with consumer expectations regarding product appearance (Naseema et al., 2021). In addition, instrumental methods such as colorimeters can provide objective and quantitative measurements of color parameters, complementing visual observation and enhancing the accuracy and consistency of appearance evaluation.

Human sensory evaluation involving taste, smell, and oral texture is another key approach for characterizing nanoemulsions, particularly in food applications where consumer acceptance is paramount. In this context, sensory evaluation focuses on food-relevant attributes including appearance, aroma, taste, bitterness, mouthfeel, turbidity, and overall acceptability (Li et al., 2021; Wang et al., 2024, Wang et al., 2024). Proper sensory evaluation should employ trained or consumer panels with appropriate sample sizes, blinding, randomization, standardized serving conditions, validated scales, and suitable statistical analysis. Abnormal changes in sensory indicators can often reflect defects in formulation. Through systematic sensory evaluation, it is possible to meet the sensory preferences of target consumer groups. In parallel, instrument-based methods including electronic noses and electronic tongues can simulate human olfaction and gustation, providing objective and quantitative analysis of aroma and flavor profiles. These instrumental techniques complement traditional sensory evaluation by enhancing reproducibility and enabling more comprehensive characterization of the aroma, taste, and appearance of nanoemulsions.

### 3.4 Interfacial structure and rheological characteristics

Interfacial structure and rheological behavior constitute critical functional characteristics governing processability, stability, and sensory performance of food-grade nanoemulsions. The interfacial layer formed by emulsifiers and biopolymers regulates droplet interactions, permeability, and responsiveness to environmental stimuli, whereas rheological properties determine flow behavior, texture, and process compatibility. Steady-shear rheometry and dynamic oscillatory analysis are routinely employed to characterize apparent viscosity, viscoelasticity, and structural integrity (Aswathanarayan and Vittal, 2019).

The rheological behavior of nanoemulsions depends largely on formulation and microstructure. Dilute nanoemulsions with low droplet volume fractions typically exhibit approximately Newtonian behavior, with viscosity remaining relatively constant across shear rates. In contrast, shear-thinning (pseudoplastic) behavior is more commonly observed in concentrated systems, flocculated dispersions, or formulations thickened with polymers or gel networks, where viscosity decreases with increasing shear rate due to progressive disruption of interdroplet interactions or structural networks (da Silva et al., 2023). Such shear-thinning behavior can be beneficial for processing and oral sensory perception in certain food applications. Rheological behavior is synergistically modulated by surfactant type, dispersed phase volume fraction, droplet size, and interdroplet interactions (Marhamati et al., 2021). Notably, biopolymer-stabilized nanoemulsions may exhibit gel-like viscoelastic behavior (G′ > G″) when the continuous phase forms an entangled network, which can enhance physical stability against coalescence and sedimentation. However, this behavior is not a general characteristic of all nanoemulsions; it is typically observed in systems with high biopolymer concentrations or in nanoemulsion-filled gel matrices. For conventional nanoemulsions with low viscosities and weak droplet interactions, the viscous modulus (G″) often predominates over the elastic modulus (G′), reflecting liquid-like behavior (Badr-Eldin et al., 2024).

Interfacial rheology has emerged as a powerful tool for characterizing the mechanical properties of droplet interfacial layers, which directly govern emulsion stability (Zhang et al., 2025). Elastic interfacial layers effectively resist droplet deformation and coalescence, whereas viscous interfaces facilitate relaxation and adaptation to mechanical stress. Recent advances demonstrate that rational design of composite interfaces (protein–polysaccharide, surfactant–biopolymer) enables synergistic enhancement of interfacial elasticity and nanoemulsion stability, representing a frontier in interfacial engineering for food-grade delivery systems (Das et al., 2020).

### 3.5 Microscopic morphology

Microscopic morphology visualization provides direct insights into droplet shape, uniformity, aggregation state, and structural integrity, complementing quantitative characterization techniques. High-resolution microscopy techniques enable nanoscale visualization of droplet microstructure, facilitating mechanistic understanding of formation, stability, and degradation mechanisms (Liew et al., 2020). Transmission electron microscopy (TEM), scanning electron microscopy (SEM), and atomic force microscopy (AFM) represent the most powerful approaches for morphological characterization (Klang et al., 2012).

Conventional TEM typically requires dilution, deposition, drying, and vacuum exposure, often with negative staining to enhance image contrast. These preparation steps may introduce artifacts such as droplet flattening, collapse, or staining-induced structural changes that can affect apparent droplet size and morphology. In contrast, cryo-TEM allows visualization of nanoemulsions in a near-native, hydrated state by rapid vitrification, preserving droplet structure without the artifacts associated with conventional TEM. Liu et al. (2024) employed TEM to characterize eugenol-hesperidin nanoemulsions, revealing spherical droplets with uniform distribution and distinct core–shell structures. SEM provides high-resolution surface morphology information but requires dehydration and conductive coating, which can induce shrinkage, cracking, or surface deformation of delicate nanostructures. Deng et al. (2022) successfully observed the microscopic morphology of silymarin nanoemulsion loaded with tea saponins as surfactants and cryoprotectants in SEM. AFM offers three-dimensional topography imaging in liquid environments, allowing characterization of interfacial structure under near-physiological conditions without extensive sample preparation (Javanshir et al., 2020). However, AFM imaging can be influenced by substrate adsorption effects, tip-sample interactions, and image-processing parameters which may alter perceived droplet dimensions and surface features. A comprehensive understanding of these methodological artifacts is essential for accurate morphological interpretation across all microscopy techniques.

Morphological characterization is indispensable for optimizing fabrication parameters, evaluating stability mechanisms, and establishing structure–function correlations guiding rational design of high-performance food-grade nanoemulsions (Mushtaq et al., 2023). Therefore, an integrated multimodal analysis combining physicochemical properties and morphological characteristics enables in-depth elucidation of emulsion stability evolution and fabrication-structure-performance relationships of nanoemulsions from a microscopic perspective.

### 3.6 Emulsion stability

Emulsion stability represents the most critical functional attribute determining shelf life, processing compatibility, and delivery performance of food-grade nanoemulsions. Nanoemulsions are kinetically stabilized colloidal systems susceptible to multiple instability mechanisms, including flocculation, coalescence, Ostwald ripening, creaming, and phase separation, driven by interfacial energy minimization and environmental stresses. Stability is governed by intrinsic factors (droplet size, zeta potential, interfacial architecture) and extrinsic factors (temperature, pH, ionic strength, light, and food matrix interactions) (Paulo et al., 2023).

Systematic stability evaluation employs multi-parameter monitoring under accelerated storage conditions (thermal, pH, ionic, and mechanical stress), tracking changes in droplet size, PDI, zeta potential, rheology, and appearance (Kampa et al., 2022). Key instability mechanisms include: 1) Flocculation: reversible droplet aggregation induced by weak interparticle forces (Onaizi, 2022); 2) Coalescence: irreversible fusion of droplets into larger particles (Paulo et al., 2023); 3) Ostwald ripening: dissolution and redeposition of smaller droplets into larger ones (Han et al., 2025); 4) Creaming/sedimentation: gravitational separation driven by density differences (Shen et al., 2024). These physical instabilities are often coupled with chemical degradation (lipid oxidation, hydrolysis) and bioactive degradation, compromising functionality. Stabilization strategies encompass formulation engineering (surfactant selection, biopolymer stabilization, oil phase optimization) and processing optimization (energy input, homogenization parameters) (Iacia et al., 2024). Natural surfactants (lecithin, proteins, polysaccharides) and composite interfaces provide sustainable stabilization through synergistic electrostatic, steric, and viscoelastic mechanisms (Fu et al., 2022). For instance, Vancolli et al. (2026) demonstrated that incorporating 1% biosurfactant enhanced the colloidal stability of *Minthostachys verticillata* essential oil nanoemulsions, with the optimized formulation showing negligible cytotoxicity toward Caco-2 cells below 750 μg/mL—a finding relevant to the safety assessment of food-grade nanoemulsions intended for oral consumption.

### 3.7 *In vitro*/*in vivo* bioactivity and bioavailability

Bioactivity retention, bioaccessibility, and bioavailability are core functional metrics for food-grade nanoemulsion carriers. Such delivery systems boost nutrient absorption via multiple pathways: improving water solubility, accelerating dissolution, shielding bioactives from degradation, tuning release behavior and promoting intestinal uptake. The evaluation of these benefits spans several distinct but interconnected endpoints, including bioaccessibility (release and solubilization during digestion), intestinal transport and pharmacokinetics (cellular uptake and systemic absorption), biological efficacy (antioxidant, antimicrobial, and anti-inflammatory activities), and toxicological safety. It is important to recognize that these endpoints represent different levels of evidence, ranging from chemical assays and in vitro cell models to ex vivo tissue studies, animal trials, food-storage tests, and ultimately human studies. These evidence levels are not equally robust; findings from chemical or cell-based assays should be considered as preliminary indicators, whereas animal studies and food-storage trials provide stronger practical evidence, and human studies remain the gold standard for validating efficacy and safety in food applications.

Simulated in vitro gastrointestinal digestion models enable rapid assessment of bioaccessibility, release characteristics, and digestive stability (Milincic et al., 2024; Nikkhah et al., 2024; Tan et al., 2025). For example, Ye et al. (2024) constructed curcumin nanoemulsions (26–129 nm, 9.53 mg/mL curcumin loading) that achieved significantly higher curcumin retention and bioaccessibility after simulated digestion compared with free curcumin. Haladyn et al. (2026) reported that whey protein-stabilized rosehip carotenoid nanoemulsions exerted more effective protective effects on carotenes than xanthophylls during simulated gastrointestinal digestion. Ke et al. (2026) demonstrated that excipient nanoemulsions increased quercetin bioaccessibility 1.73-fold using the INFOGEST model.

Beyond bioaccessibility, cellular uptake studies and *in vivo* pharmacokinetic trials provide evidence of intestinal transport and systemic bioavailability. Ye et al. (2024) reported that curcumin nanoemulsions exhibited significantly higher cellular uptake than free curcumin in Caco-2 models. Ke et al. (2026) further validated the bioavailability-promoting effect in rat *in vivo* trials, showing that nanoemulsion co-administration increased quercetin bioavailability by 46.6% and systemic antioxidant capacity by 1.47-fold (P < 0.05).

The enhanced bioaccessibility (the fraction released from the food matrix and solubilized during digestion) and bioavailability (the fraction that reaches systemic circulation) of nanoencapsulated bioactives are expected to translate into improved biological efficacy. Mehidi et al. (2025) fabricated *Satureja battandieri* essential oil nanoemulsions (PDI < 0.3) that produced stronger antibacterial suppression against common foodborne pathogens and enhanced antioxidant capacity compared with the free oil. The study also reported anti-inflammatory activity in murine models (64% edema inhibition vs 32% for pure oil), which is discussed here as indirect evidence of enhanced bioactivity conferred by nanoencapsulation. Haladyn et al. (2026) assessed the biosafety of rosehip carotenoid nanoemulsions, providing preliminary cytotoxicity data relevant to food application. Together, these efficacy and safety endpoints complement the bioaccessibility and transport data discussed above, collectively supporting the functional potential of food-grade nanoemulsions while highlighting the need for more systematic *in vivo* toxicological evaluation.

It should be noted that direct comparison across these studies is limited by differences in formulations, experimental models, and assay conditions, highlighting the need for more standardized evaluation frameworks to fully establish the translational potential of food-grade nanoemulsions.

### 3.8 Advanced characterization methodologies

In addition to the fundamental physicochemical characteristics described above, advanced analytical techniques have been increasingly employed to reveal the interfacial, structural, and dynamic properties of nanoemulsions. For instance, interfacial tension, a key indicator of emulsification efficiency and droplet stability, is generally measured using liquid-liquid tensiometric methods such as pendant-drop, spinning-drop, or Wilhelmy-plate techniques (Wang et al., 2025). Fourier-transform infrared (FT-IR) spectroscopy identifies functional groups and molecular interactions (e.g., hydrogen bonding) in nanoemulsion components, providing qualitative compositional information; however, spectral overlaps and strong water absorption in the mid-IR region may complicate interpretation (Song et al., 2025). Differential scanning calorimetry (DSC) measures thermal transitions (melting, crystallization, glass transition) of lipid phases, offering insights into the physical state of encapsulated oils; results are sensitive to sample preparation, heating rate, and thermal history (Ghadermazi et al., 2026). Nuclear magnetic resonance (NMR) spectroscopy provides molecular-level information on chemical structure and diffusion behavior without extensive sample destruction, though sensitivity may be limited in diluted systems and spectral assignment can be challenging in complex food matrices (Bautista-Hernández et al., 2025; Hore et al., 2023). Molecular dynamics simulation computationally models molecular interactions and interfacial behavior at atomic resolution, offering mechanistic insights, but its predictive accuracy depends on force-field parameters and simulation time scales. X-ray photoelectron spectroscopy (XPS) is a surface-sensitive technique that provides elemental composition and chemical state information of nanoemulsion interfaces, enabling analysis of emulsifier adsorption layers and surface chemistry. Its application requires dried samples and ultra-high vacuum, which may alter native interfacial structures; nevertheless, it offers complementary information to FT-IR and NMR for understanding interfacial phenomena in nanoemulsions. Together, these advanced methods provide complementary information on the structural, thermal, and dynamic properties of nanoemulsions, contributing to a more comprehensive understanding of their stability and functional performance.

To provide an integrated evaluation of food-grade nanoemulsions, the evidence discussed in this review can be organized into a formulation-to-translation framework (Fig. 3). Formulation composition and fabrication conditions jointly determine droplet and interfacial characteristics, which subsequently influence stability under food-processing and storage conditions. For orally consumed systems, these properties further affect gastrointestinal behavior and bioaccessibility. However, favorable physicochemical properties alone do not ensure practical applicability. Sensory acceptance and safety must also be considered before industrial translation. Accordingly, industrial readiness should be evaluated by integrating processing stability, gastrointestinal performance, sensory quality, safety, scalability, reproducibility, energy demand, and production feasibility. This framework emphasizes that evidence at each stage should be considered collectively rather than using individual parameters, such as droplet size or encapsulation efficiency, as independent indicators of application potential.

**Fig. 3:**
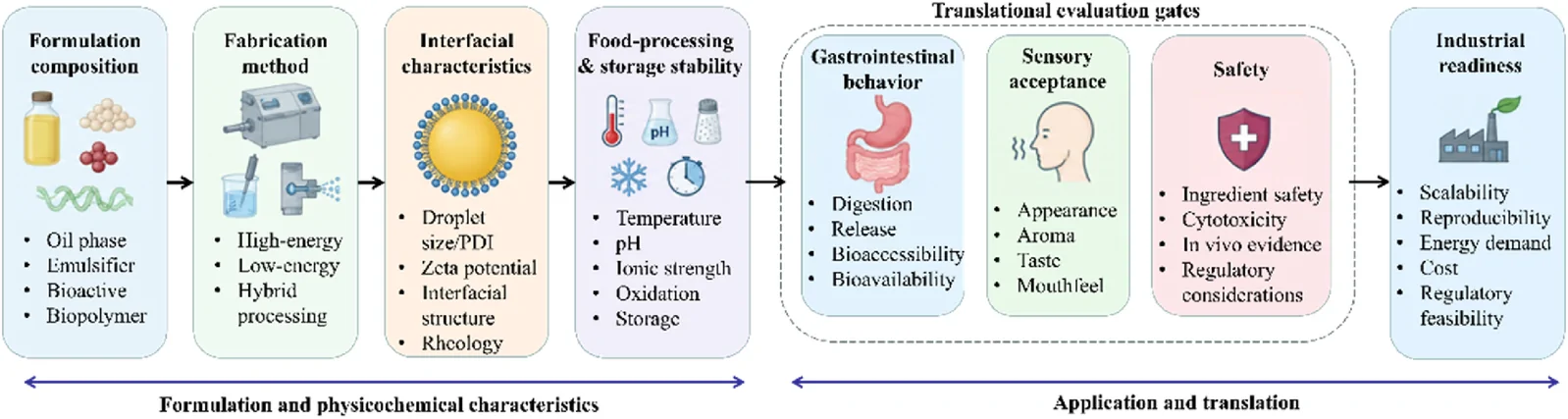
Integrated framework for evaluating food-grade nanoemulsions from formulation design to industrial translation.

## 4 Applications of nanoemulsions in the food industry

Food-grade nanoemulsions represent delivery platforms for next-generation functional foods, offering versatile capabilities for bioactive protection, solubility enhancement, controlled release, and quality improvement. Their unique physicochemical properties (nanoscale size, high stability, improved bioavailability) enable versatile applications across three strategic food industry sectors: food quality improvement (covering both direct incorporation into food matrices and surface coatings for postharvest preservation), novel food packaging films (including both functional preservative films and intelligent freshness-indicating packaging), and manufacturing functional foods. The applications of food-grade nanoemulsions are presented in Fig. 4.

**Fig. 4:**
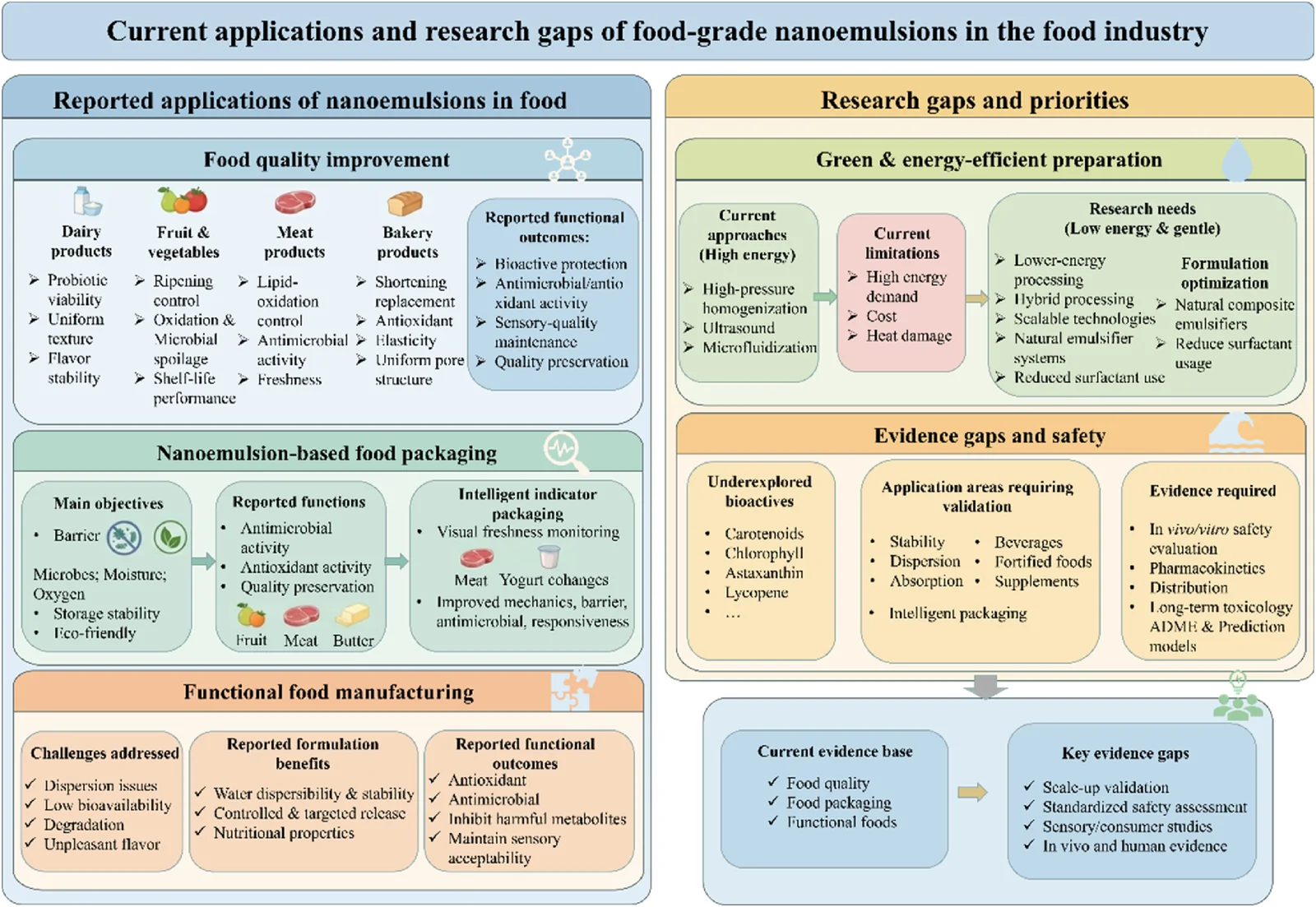
Multifunctional applications, challenges and future development trends of nanoemulsions in food field.

### 4.1 Food quality improvement

Nanoemulsion technology enables multifaceted food quality improvement through texture modification, flavor enhancement, shelf-life extension, and nutritional fortification. Relevant studies on nanoemulsions for food quality improvement are summarized in Table 3. The functional effects of nanoemulsions are mainly associated with improved dispersion and protection of encapsulated bioactives and their controlled release, which may enhance antimicrobial and antioxidant performance in food systems.

**Table 3:** Application of nanoemulsion in food quality improvement.

Applied food matrix	Nanoemulsion formulation	Functional benefits & effects	Reference
Dairy product	Spearmint, lemongrass, clove, and cinnamon essential oils	Maintained probiotic viability (>8 log CFU/g) during 28-day refrigerated storage compared with 6.5 log CFU/g in the free-oil control, while retaining acceptable sensory quality. The main benefit was therefore probiotic protection rather than direct shelf-life extension	Salama et al. (2022)
	Nanoemulsified *Planiliza abu* protein hydrolysate (<10 kDa)	Enhanced antioxidant activity, LAB survival, and water-holding capacity while reducing syneresis during 21-day cold storage, indicating simultaneous effects on microbial viability and physical quality	Vakili et al. (2024)
	Oregano essential oil	Improved antioxidant capacity and reduced protein and lipid oxidation at 4 °C, extending shelf life by approximately 12 days. This preservation effect reflects both oxidative protection and storage conditions	Hamouda et al. (2025)
	Citral and lemongrass oil	Reduced microbial contamination by > 3 log CFU/g during 14-day storage and showed preliminary safety at the tested concentrations. The study primarily supports antimicrobial efficacy, with only limited safety evidence	Cunha et al. (2026)
Fruit and vegetable product	Hexanal and trans-2-hexenal	Combined nanoemulsion and UHPH treatment reduced microbial counts and extended apple-juice shelf life from 7 to 21 days without compromising sensory quality, suggesting a combined formulation–processing effect	Patrignani, Lorenzo Siroli, Giacomo Braschi, and Rosalba Lanciotti (2020)
	Acetic acid-(1H-indole-3-ethyl) ester (AIE)	When incorporated into a chitosan coating, the nanoemulsion reduced fungal decay and weight loss and maintained firmness during 28-day storage, indicating that preservation resulted from both the active nanoemulsion and the coating matrix	Gong et al. (2026)
	Caraway essential oil and chitosan	Reduced microbial growth and weight loss and extended banana shelf life from 6 to 12 days at ambient temperature. The reported effect is therefore specific to both the coating system and ambient-storage conditions	Das et al. (2023)
	Methyl salicylate (MeSA)	Delayed potato sprouting by approximately 9 days while maintaining soluble sugar and starch contents. Unlike microbial shelf-life studies, this application mainly targets physiological deterioration	Hou et al. (2026)
	Carnosic acid	Reduced grape decay and weight loss during cold storage and showed strong antifungal activity. The preservation effect was evaluated mainly through fungal inhibition and postharvest quality rather than a single shelf-life endpoint	Ye et al. (2026)
	Zein-chitosan-salicylic acid	Reduced weight loss, maintained firmness, enhanced antioxidant capacity, and extended mango shelf life to 21 days. The effect reflects combined regulation of physical quality and oxidative stress	Fan et al. (2026)
Meat product	Chitosan-gum Arabic-MCT stabilized thyme essential oil	Reduced TVB-N and TBARS during meat storage while maintaining colloidal stability. This study mainly demonstrated delayed chemical deterioration rather than reporting a directly comparable shelf-life value	Snoussi et al. (2022)
	Ginger essential oil	In duck meat stored at 4 °C, the nanoemulsion reduced microbial growth and lipid oxidation and extended shelf life from 14 to 35 days. The magnitude of this effect is dependent on the meat matrix, formulation, and shelf-life criteria	Chen et al. (2025)
	Composite essential oil nanoemulsions (CEO-NE) via covalent bonding & compositional synergy	In refrigerated hairtail, the formulation inhibited *E. coli* and *S. aureus* and maintained product quality for >12 days. Direct comparison with the 35-day value reported for duck meat is limited by differences in food matrix, active composition, and shelf-life definition	Wang et al. (2026)
	Eugenol	In fermented sausage, the nanoemulsion suppressed histamine-producing bacteria, stabilized the microbial community, and reduced histamine accumulation. The major endpoint was biogenic-amine control rather than conventional refrigerated shelf-life extension	Li et al. (2026)

In dairy products, nanoemulsions enhance probiotic survival, texture uniformity, and flavor stability. Nanoemulsions of plant essential oils (such as spearmint, lemongrass, clove, and cinnamon) (30 nm) have been proven to remarkably improve the viability of *Lactobacillus acidophilus*, *Lactobacillus helveticus*, and *Bifidobacterium* in stirred yogurt, while imparting natural flavors to the products (Salama et al., 2022). Additionally, Cunha et al. (2026) reported that stable citral and lemongrass oil nanoemulsions exerted strong antibacterial activity against *Staphylococcus aureus* and effectively lowered microbial contamination in ricotta cheese, with zebrafish *in vivo* assays providing preliminary safety data. For fruit and vegetable products, nanoemulsions can effectively inhibit ripening, oxidative degradation, and microbial spoilage. Nanoemulsions formulated with hexanal and trans-2-hexenal by high pressure homogenization exhibited potent antimicrobial activity and improved the quality of apple juice due to their organoleptic compatibility (Patrignani et al., 2020a, Patrignani et al., 2020b). Q. H. Gong et al. (2026) reported that corn oil-based acetic acid ester (2%, w/v) nanoemulsion (152 ± 2.35 nm) with favorable colloidal stability strongly suppressed *Penicillium expansum* and improved apple preservation over 28 days when incorporated into chitosan coatings. Rapeseed oil-chitosan nanoemulsions (105.0 ± 8.4 nm) reduced banana weight loss, inhibited oxidative stress, and preserved color, taste, and nutritional value during storage (Das et al., 2023). Moreover, Hou et al. (2026) reported that methyl salicylate nanoemulsion successfully delayed potato sprouting by 9 days and preserved tuber quality via sustained release of the active inhibitor. Ye et al. (2026) reported that the carnosic acid nanoemulsion (19.42 nm, PDI = 0.09) exhibited enhanced thermal stability and antifungal activity against mycelial growth and spore germination of *Colletotrichum viniferum* (EC_50_ values of 47.43 and 23.77 mg/L), which effectively controlled grape anthracnose and maintained fruit quality under cold storage. Fan et al. (2026) also reported that a salicylic acid nanoemulsion encapsulated within a zein–chitosan composite shell (247 nm, +30.3 mV) can preserve postharvest mango quality, owing to the synergistic regulation of oxidative stress and hormone signaling pathways. In meat products, nanoemulsions outperform pure essential oils in shelf-life extension and bioactivity enhancement, overcoming the drawbacks of poor water solubility, high volatility and low long-term stability of raw oils (da Silva, do Rosario, Weitz and Conte-Junior, 2022). Thyme essential oil-based O/W nanoemulsions formulated with 2% (w/w) thyme essential oil in medium-chain triglycerides (MCT) present outstanding preservation efficacy for meat. During storage, the nanoemulsion-treated group showed non-significant slight increases in pH, total volatile basic nitrogen (TVB-N), thiobarbituric acid reactive substances (TBARS) and shear strength; significant differences between control and treated samples further validated its favorable preservation effect (Snoussi et al., 2022). Wang et al. (2026) indicated that the composite essential oil (rosemary and thyme) nanoemulsion can extend hairtail (*Trichiurus lepturus*) storage life beyond 12 days. Additionally, Li et al. (2026) reported that eugenol nanoemulsion (318.6 nm, PDI 0.227) suppressed histamine-producing spoilage bacteria in fermented sausage by downregulating histidine decarboxylase genes. Its sustained antibacterial performance stabilized microbial communities and markedly reduced histamine accumulation. For bakery products, Uday Bagale et al., 2022a, Bagale et al., 2022b reported that curcumin nanoemulsion substitution for margarine improved bread sensory profiles, crumb elasticity, and pore uniformity.

Overall, the effectiveness of nanoemulsions in food quality improvement is highly matrix-dependent. In dairy products, the main benefits involve probiotic viability, oxidative stability, and texture, whereas fruit and vegetable applications primarily target fungal decay, weight loss, ripening, and postharvest deterioration. In meat products, antimicrobial and antioxidant effects are more directly associated with shelf-life extension. However, these values should not be directly compared because the studies used different food matrices, active compounds, formulations, storage conditions, and shelf-life criteria. Thus, the practical performance of nanoemulsions should be evaluated according to the specific deterioration mechanism and food matrix rather than droplet size or shelf-life extension alone.

### 4.2 Novel food packaging films

Food packaging primarily functions to shield food from microbial invasion, moisture and oxygen exposure, thereby inhibiting quality deterioration and improving storage stability. Nevertheless, the extensive application of non-degradable plastic packaging has raised serious environmental concerns. Plastic packaging accounted for approximately 36% of global plastic production in 2015 and generated nearly half of total plastic waste because of its relatively short service life. Food, beverage, and tobacco packaging has also been reported to constitute more than 60% of global beach litter. These environmental concerns have stimulated increasing interest in biodegradable and intelligent food-packaging systems, including biopolymer-based pH-responsive films for real-time freshness monitoring (Kiani-Salmi et al., 2025). Accordingly, biodegradable and edible functional films have attracted increasing attention as alternatives to conventional plastic packaging. In recent years, the development and application of degradable and edible functional films have become a major research focus in food packaging (Daud et al., 2026; Hashim et al., 2024; Huang et al., 2026; Shen et al., 2023). Relevant studies on nanoemulsions for novel food-packaging films are summarized in Table 4.

**Table 4:** Application of nanoemulsion in food packaging films.

Nanoemulsion formulation	Delivery system type	Film properties	Functional benefits & effects	Reference
Curcumin and orange essential oil	Nanoemulsion-loaded film	Improved water-vapor barrier, tensile strength, flexibility, and oil retention	Inhibited microbial growth and prolonged fruit preservation. The reported benefit mainly concerns active preservation rather than mechanical or indicator performance	Rashid et al. (2023)
Camellia Oil-Bacterial Cellulose Nanocrystalline Pickering emulsion	Pickering emulsion-based film	Improved hydrophobicity, UV shielding, mechanical strength, thermal stability, and water-vapor barrier	Enabled visual monitoring of yogurt freshness through color changes. This system primarily functions as an intelligent indicator rather than as a conventional shelf-life-extending film	Li et al. (2023)
Cinnamon essential oil	Nanoemulsion-loaded film	Enhanced thermal stability, UV barrier, water resistance, and sustained-release properties	Delayed pork deterioration while providing color-based freshness indication, demonstrating combined active-preservation and intelligent-monitoring functions	Ran et al. (2023)
Essential oil and polyphenol	Nanoemulsion-coated film		Improved packaging performance and prolonged food preservation, although the reported effects reflect both film modification and active-compound release	Panwar et al. (2024)
Citral	Nanoemulsion-treated coating		Reduced surface microbial contamination on fresh-cut carrots. The principal endpoint was short-term microbial control rather than film mechanical performance	Song et al. (2024)
Soybean protein/lavender essential Oil	Nanoemulsion-loaded film	Improved mechanical properties, water-vapor barrier, and film structural uniformity	Inhibited bacterial growth and prolonged the freshness of *Volvariella volvacea*, indicating a preservation effect that depends on both the protein film matrix and essential-oil activity	Khan et al. (2024)
Chitosan/pullulan composite edible coating incorporated with 2% thyme essential oil	Nanoemulsion-coated film	Improved water-vapor barrier and tensile properties with sustained-release behavior	Reduced fungal growth, weight loss, and decay in strawberries and extended refrigerated shelf life to 12 days at 2 °C. The result is specific to the coating composition and low-temperature storage conditions	Chen et al. (2025)
Flaxseed oil nanoemulsion loaded with ferulic acid	Nanoemulsion-loaded film	Reduced water-vapor and oxygen permeability; enhanced tensile strength, flexibility, and surface hydrophobicity	Prolonged pork preservation, with the effect mainly attributed to active antioxidant and preservation functions rather than freshness indication	Wen et al. (2025)
Gelatin film incorporated with *Satureja kermanshahensis* essential oil nanoemulsion and selenium nanoparticles	Nanoemulsion-loaded film	Improved mechanical strength; reduced water-vapor permeability and swelling; enhanced thermal stability with SeNP incorporation	Provided antioxidant, antibacterial, and UV-barrier functions. In contrast to studies focused mainly on shelf life, this work primarily demonstrated multifunctional film performance	Roshandel et al. (2026)
CMC/sodium alginate composite films loaded with turmeric essential oil nanoemulsion & gallic acid	Nanoemulsion-loaded film	Enhanced mechanical and barrier properties	Enhanced antioxidant and antibacterial activity and reduced grape weight loss, linking improvements in film properties with postharvest preservation	Shah et al. (2026)
Hempseed protein-stabilized ginger essential oil Pickering nanoemulsion + aronia anthocyanin loaded chitosan/carrageenan film	Pickering nanoemulsion-based film	Optimized mechanical strength and barrier performance	Combined antioxidant and antibacterial activity with pH-responsive color changes, indicating simultaneous active-packaging and freshness-monitoring functions	Talebi et al. (2026)
Chitosan composite films incorporated with *Litsea cubeba* essential oil nanoemulsion	Nanoemulsion-loaded film	Enhanced oxygen barrier properties	Reduced lipid oxidation and mold growth in butter while improving oxygen-barrier performance, showing that preservation was closely associated with both active compounds and barrier properties	Zhang et al. (2026)
κ-carrageenan gel films loaded with clove essential oil nanoemulsion	Nanoemulsion-loaded film	Improved flexibility; strong UV barrier	Suppressed spoilage microorganisms and extended cold-storage shelf life to 10 days while also improving UV-barrier performance. The reported outcome therefore combines preservation and film-function enhancement	Liu et al. (2026)

Numerous studies have confirmed that incorporating volatile plant essential oil nanoemulsions into degradable polysaccharide-based, protein-based or composite film matrices can effectively inhibit microbial growth on the surface of fruits and vegetables, maintain their quality during storage, and significantly extend their shelf life (Liu et al., 2026; Roshandel et al., 2026; Shah et al., 2026; Xie et al., 2026). Additionally, nanoemulsion-embedded preservative films have also been investigated for the preservation of meat products, butter and other fatty foods. Owing to their superior antioxidant and antibacterial activities, such films can effectively delay sensory quality deterioration, inhibit lipid oxidation, and thereby markedly extend the shelf life of food products (Ashraf et al., 2025; Ayoub et al., 2025; Rashid et al., 2024; Wen et al., 2025; Zhang et al., 2026). This strategy provides a potential alternative to conventional plastic food packaging, though its environmental performance requires further evaluation. In packaging systems, active compounds may gradually diffuse from nanoemulsion-containing films toward the food surface or headspace, with their migration and release influenced by film composition, temperature, humidity, and food-matrix properties. From a circular-economy perspective, future nanoemulsion-based packaging should also consider material recovery, recyclability or compostability, and compatibility with closed-loop waste-management systems.

In recent years, intelligent color-indicating packaging represents another emerging research direction for monitoring food freshness (Ding et al., 2025; Wu et al., 2025; Zhai et al., 2024). Ran et al. (2023) developed cinnamon essential oil nanoemulsions stabilized by oxidized cellulose nanofibers, which were combined with curcumin to prepare functional films for pork packaging. The films not only delayed pork quality deterioration but also enabled visual monitoring of freshness via a color transition from bright brown to saddle brown. Furthermore, Li et al., 2023, Li et al., 2023 constructed a multifunctional intelligent packaging system by incorporating bacterial cellulose nanocrystal-stabilized tea oil Pickering emulsion into an alginate-anthocyanin matrix. Applied in yogurt preservation, this packaging presented distinct color variations to enable intuitive freshness evaluation. Additionally, Talebi et al. (2026) fabricated hempseed protein nanoparticle-stabilized ginger essential oil Pickering nanoemulsions, which together with pH-sensitive aronia anthocyanin were integrated into chitosan/carrageenan edible films to simultaneously strengthen mechanical, barrier, antioxidant and antibacterial performance while endowing the packaging films with ammonia-responsive color-changing a freshness-indicating functions. In edible and intelligent packaging systems, pH can also serve as an important indicator of food quality and freshness because pH changes may reflect microbial growth and biochemical deterioration during storage. For hydrophilic biopolymer-based packaging films, moisture sensitivity remains an important practical limitation. High humidity may increase water uptake and plasticization of the film matrix, thereby weakening mechanical and water-barrier properties; this issue may be mitigated through hydrophobic modification, cross-linking, or multilayer/composite film design. Other parameters, such as color, lipid oxidation, microbial counts, texture, and sensory attributes, can provide complementary information for evaluating food deterioration and safety. However, digital monitoring of intelligent packaging may face practical challenges, including variations in illumination and imaging devices, environmental interference, signal drift, and differences among food matrices. These limitations can be reduced through standardized image-acquisition conditions, reference-based calibration, periodic sensor recalibration, and validation using multiple food-quality indicators. Taken together, nanoemulsion-based packaging should be evaluated as an integrated film–active compound–food system rather than solely by its antimicrobial activity. For active packaging, improved preservation must be balanced against changes in mechanical strength, barrier properties, moisture sensitivity, and controlled migration or release of active compounds. For intelligent packaging, reliable color response additionally requires resistance to humidity, illumination variation, signal drift, and differences among food matrices. Therefore, future comparisons should distinguish between preservation performance, film functionality, and sensing capability, while also considering cost, scalability, and food-contact safety.

### 4.3 Manufacturing functional foods

Growing public awareness of health has boosted consumer demand for functional foods with nutritional and bioactive benefits. Nevertheless, the applications of most natural bioactive compounds are limited by poor dispersibility, low oral bioavailability, high susceptibility to degradation during processing and storage, and undesirable flavors, limiting their practical food applications. Nanoemulsions have been recognized as an effective approach to address these drawbacks (Bravo-Alfaro et al., 2025; Yan et al., 2025). By encapsulating bioactives within nanoscale oil-in-water droplets, nanoemulsions ameliorate their water dispersibility and environmental stability while enabling controlled and targeted release, offering a viable technical route for developing functional food products (Niroula et al., 2024; Tan et al., 2023). Relevant studies on nanoemulsions for manufacturing functional foods are summarized in Table 5.

**Table 5:** Application of nanoemulsion in manufacturing functional foods.

Nanoemulsion formulations	Functional benefits & effects	References
Cinnamon essential oil	Combined with ascorbic acid, the nanoemulsion inhibited PPO activity, reduced enzymatic browning, improved suspension stability, and delayed nutrient loss in cloudy apple juice. The main evidence relates to processing and storage quality rather than nutritional bioavailability.	Xu et al. (2020)
β-Carotene	Improved β-carotene delivery and bioavailability, supporting its use in functional food and beverage systems. This outcome focuses primarily on nutrient delivery rather than product shelf life or sensory quality.	Mehmood et al. (2021)
Alginate/zein	Enabled controlled glucose release in fruit-flavored products, indicating potential for modulating nutrient release rather than simply improving physicochemical stability.	Razavi et al. (2021)
Curcumin	In bread, the nanoemulsion increased antioxidant activity and improved crumb structure, elasticity, and sensory properties when partially replacing margarine. The benefit therefore combines nutritional and technological functions.	Bagale et al. (2022)
Essential oil (pennyroyal and Gijavash)	In pasteurized yogurt drinks, the nanoemulsion improved preservation while maintaining acceptable sensory properties, showing that antimicrobial functionality must be balanced with consumer acceptability.	Habibvand et al. (2023)
Linseed oil-EGCG	In salad dressing and baked mooncakes, the nanoemulsion enhanced antioxidant and antibacterial activities and reduced undesirable chemical changes. The magnitude of these effects is food-matrix and processing dependent.	Xie et al. (2024)
Clove oil	Enabled encapsulation and delivery of quercetin and vitamin B for functional-food applications. The available evidence primarily demonstrates delivery capability rather than direct *in vivo* efficacy.	Srivastava et al. (2025)
Basil seed hydrogel-based saffron petal & vitamin D_3_	Maintained colloidal stability and enabled vitamin D3 fortification of dairy powder, while antioxidant retention, vitamin preservation, and sensory quality varied with drying conditions. This highlights processing conditions as a major determinant of final functionality.	Hashemi et al. (2026)

Xie et al. (2024) prepared optimized linseed oil-EGCG nanoemulsions. Incorporated into salad dressings and baked mooncakes, the nanoemulsions improved sensory quality, enhanced antioxidant and antibacterial performance, inhibited acrylamide production and unsaturated fatty acid degradation, facilitating the development of nutrient-fortified functional foods. Xu et al. (2020) verified that cinnamon essential oil nanoemulsion combined with ascorbic acid fully suppressed PPO activity, inhibited enzymatic browning, improved suspension property and retarded nutrient loss of cloudy apple juice during storage. Furthermore, Habibvand et al. (2023) optimized essential oil nanoemulsions (pennyroyal and Gijavash) as natural preservatives for pasteurized yogurt drinks, which improved colloidal stability, as indicated by the zeta potential and antioxidant capacity, inhibited microbial proliferation, and maintained favorable sensory acceptability, offering a green alternative to synthetic food preservatives. Additionally, Bagale et al., 2022a, Bagale et al., 2022b demonstrated that curcumin nanoemulsions fabricated via ultrasonic treatment could replace margarine in bread production. The incorporation of curcumin nanoemulsions significantly improved the antioxidant capacity, crumb elasticity and pore uniformity of bread while maintaining acceptable sensory quality, thereby enhancing the nutritional and textural properties of baked products. Moreover, Hashemi et al. (2026) fabricated stable basil seed hydrogel nanoemulsions co-encapsulating saffron petal bioactives and vitamin D_3_ to fortify functional milk powder by conventional spray drying, and the resulting fortified powder contained 115.63 IU vitamin D_3_, almost 20 times the content found in unfortified spray-dried milk powder.

Collectively, nanoemulsions can contribute to functional-food development through several distinct routes, including nutrient fortification, protection of oxidation-sensitive compounds, replacement of conventional ingredients, and improvement of sensory or processing properties. However, these technological benefits should be distinguished from nutritional efficacy. Improved stability, antioxidant activity, or in vitro delivery does not necessarily demonstrate enhanced *in vivo* bioavailability or health effects. In addition, processing conditions such as heating, baking, storage, and drying may strongly influence the retention and functionality of encapsulated bioactives. Therefore, future studies should integrate processing stability, sensory acceptability, digestive behavior, and *in vivo* validation when assessing the practical value of nanoemulsion-fortified foods.

## 5 Future research directions for nanoemulsions

### 5.1 Green and energy-efficient preparation of nanoemulsion

High-energy homogenization (high-pressure homogenization, conventional ultrasonication) remains a mainstream approach for industrial nanoemulsion fabrication owing to controllable particle size and narrow distribution, yet intrinsic drawbacks including excessive power consumption, costly processing equipment and temperature increase during homogenization severely restrict green industrialization. High-pressure homogenization can produce nanoemulsions below 200 nm (Sneha & Kumar, 2022), but continuous pressurization elevates system temperature, triggering thermal denaturation of heat-labile components such as natural vitamins, plant enzymes and unsaturated lipids (Seung Jun Choi and McClements, 2020). Conventional high-intensity ultrasonic homogenization relies on cavitation to split oil droplets, whereas excessive acoustic energy easily induces protein denaturation, polysaccharide chain scission and lipid peroxidation, impairing emulsion physicochemical stability and encapsulated bioactivity (Li et al., 2021; Qayum et al., 2023).

Emerging low-energy spontaneous emulsification, thermo-induced nano-emulsification and flash nanoprecipitation (FNP) serve as prospective green alternatives, whereas a single low-energy technique commonly requires excessive surfactant dosage and is susceptible to ambient pH and temperature fluctuations, restricting large-scale continuous production. Accordingly, synergistic compounding of partial low-energy spontaneous emulsification with mild high-energy treatment has become a core developmental trend: this hybrid route cuts total energy input and processing temperature increment simultaneously, effectively preserving the structural integrity of thermosensitive bioactives without sacrificing nanoemulsion droplet uniformity. Besides the existing combined homogenization strategy, microfluidic continuous synthesis and non-equilibrium surfactant-driven spontaneous emulsification newly developed in recent years further lower unit energy consumption and reduce surfactant addition, representing cutting-edge green preparation directions for food-grade nanoemulsions.

In terms of emulsifier formulation, synthetic surfactants (Tween series, SDS) feature rapid interfacial film formation and outstanding emulsifying efficiency, yet potential biological toxicity, impaired gastrointestinal bioavailability of encapsulated nutrients and post-discard environmental contamination limit their application in clean-label food products (Castor, do Nascimento and Gorlach-Lira, 2024; Dai et al., 2023; Shen et al., 2026). Natural food-grade surfactants including lecithin, plant proteins and food polysaccharides have dominated green formulation research due to full biodegradability and low *in vivo* toxicity (Lin et al., 2024; Liu et al., 2025; Xu et al., 2024; Yeboah et al., 2025). Lecithin alone can improve carotenoid and plant extract solubility and oral bioavailability via compact interfacial layer construction (Flores-Andrade et al., 2021); a composite system comprising xanthan gum, whey protein and plant-derived saponin achieves synergistic stabilization via electrostatic interaction and steric hindrance, optimizing emulsion storage stability and sensory performance synchronously, matching the formulation requirement of beverage and baked food (Tu et al., 2025). Nevertheless, natural emulsifiers used alone typically require higher concentrations and are sensitive to ionic strength and thermal processing, restricting their standalone industrial use.

Future preparation optimization should center on three core targets: energy-saving compound homogenization technology development, all-natural composite emulsifier screening, and reduction of total surfactant dosage. Optimizing process parameters of hybrid high/low-energy preparation and developing multi-component natural emulsifier blends can jointly balance the trade-offs among production cost, product safety and environmental friendliness, accelerating the broader adoption of nanoemulsions in functional food and oral nutrient delivery sectors. Beyond laboratory optimization, industrial translation requires attention to batch reproducibility, equipment fouling and cleaning, throughput, cost per unit volume, energy consumption, continuous processing, and validation in realistic food matrices. These factors are rarely reported but are critical for assessing commercial potential.

### 5.2 Broadened nanoemulsion application

Current research on nanoemulsions mainly focuses on encapsulating essential oils, polyphenols and vitamins to improve their water solubility, chemical stability and gastrointestinal bioavailability. Although several studies have investigated the nanoencapsulation of lipophilic natural pigments (e.g., astaxanthin, carotenoids), these investigations remain largely at the laboratory scale. Industrial-scale validation, long-term stability, and systematic *in vivo* safety data are still lacking, forming a distinct translational gap. These pigments exhibit potent bioactivities (Zhang et al., 2024a; Zhang et al., 2024b), yet their hydrophobicity and degradation susceptibility restrict their direct application (Zhang et al., 2020, Zhang et al., 2020). Nanoemulsion delivery systems can address these limitations; however, bridging the translational gap requires research focusing on industrial-scale fabrication, long-term stability, and *in vivo* validation. In addition to these technical challenges, several regulatory and safety aspects must also be addressed for successful industrial translation, including regulatory classification, acceptable daily intake limits, residual solvent compliance, packaging migration, chronic exposure assessment, protein emulsifier allergenicity, gut microbiome effects, consumer perception and labeling, as well as environmental fate and biodegradability. These aspects remain underexplored in the current literature and will be critical for bridging the gap between laboratory development and real-world food applications.

From a broader sustainability perspective, food-grade nanoemulsions may contribute to the Sustainable Development Goals (SDGs) by improving food preservation, reducing food loss and waste, and promoting more efficient resource utilization (Manzoor et al., 2024). Nanoemulsion-based active and intelligent packaging may further support sustainable food production by extending shelf life and monitoring or maintaining food quality (Bahramian et al., 2025). These advances may contribute to sustainable consumption and production and climate-change mitigation, while their integration with automated processing and monitoring technologies may support more intelligent food manufacturing. However, these potential benefits require further validation through life-cycle assessment, energy-use analysis, and industrial-scale studies.

Stabilization via binary protein-polysaccharide composite systems further elevates pigment retention during thermal sterilization and long-term cold storage, making such nanoemulsions highly compatible with large-scale industrial food manufacturing (Ke et al., 2025). Additionally, natural pigment-loaded nanoemulsions can act as pH-responsive colorimetric indicators incorporated into edible packaging films. Compared with directly adding free pigments to film matrices, nanoemulsion encapsulation homogenizes pigment dispersion and enhances the color-changing stability of anthocyanins and carotenoids during shelf storage. This unique responsive characteristic expands the application potential of intelligent edible packaging and provides robust technical strategies for real-time food freshness monitoring and consumer safety assurance.

Furthermore, the bioactivity assessment of nanoemulsions largely relies on in vitro simulated gastrointestinal digestion and chemical antioxidant assays. In contrast, systematic *in vivo* evidence covering pharmacokinetics, tissue biodistribution and long-term toxicological evaluation remains insufficient. Follow-up research should integrate Caco-2 monolayer permeation tests, *in vivo* metabolic tracing in animal models and short/long-term feeding trials to systematically clarify the absorption, distribution, metabolism and excretion (ADME) profiles of encapsulated natural bioactive ingredients, establish reliable quantitative *in vitro-in vivo* prediction models, and fill the gaps in systematic safety evaluation for nanoemulsions.

In summary, the development of nanoemulsions loaded with natural bioactive compounds has considerable scientific significance and industrial transformation prospects. By optimizing green low-energy nanoemulsion fabrication techniques, constructing all-natural binary composite stabilizer systems, and combining multi-dimensional in vitro characterization with hierarchical *in vivo* efficacy and safety verification, nanoemulsion technology will evolve into a core technical platform to achieve high-value utilization of bioactive ingredients. This technology facilitates the upgrading of clean-label functional foods and mitigates industrial reliance on synthetic colorants and chemical preservatives. Both low-energy self-emulsification and high-shear high-energy emulsification exhibit respective strengths for different industrial production scenarios, requiring targeted optimization according to product demands. The core bottlenecks restricting food-grade nanoemulsion development and prospective research directions are summarized in Fig. 4.

## 6 Conclusion

This review critically summarizes recent advances in food-grade nanoemulsions in fabrication technologies, multi-dimensional characterization and food functional applications. High-energy emulsification achieves uniform nanodroplet formation but is constrained by high energy consumption, thermally induced degradation of bioactive compounds and high equipment costs. In contrast, low-energy emulsification offers green and low-cost fabrication, yet it is limited by poor formulation stability and environmental susceptibility. The combination of hybrid high-/low-energy emulsification and natural interfacial engineering has shown potential as a strategy to balance production efficiency, energy consumption and product safety, though systematic comparative studies under industrial conditions remain limited. Multiple characterization techniques, including particle size analysis, zeta potential determination, rheological testing and interfacial molecular characterization, provide reliable technical support for evaluating the colloidal stability and structural durability of nanoemulsions under practical food processing and storage conditions.

Owing to their unique nanoscale properties and excellent encapsulation performance, nanoemulsions have been shown to improve the water solubility and environmental stability of lipophilic bioactive substances in laboratory studies. Enhanced oral bioavailability has been reported for specific formulations in vitro digestion models and some animal studies, though evidence from human trials remains limited. Such delivery systems exert versatile functions in food preservation, sensory optimization and nutritional fortification in laboratory-scale studies, suggesting potential for reducing dependence on synthetic additives, though industrial-scale validation and comprehensive safety assessments are still needed. Importantly, this review identifies a prominent research gap in the nanoencapsulation and industrial application of lipophilic natural pigments (astaxanthin, lycopene and chlorophyll), whose biological potential has not been fully exploited in current food research.

Despite abundant in vitro studies on formulation optimization and functional evaluation, existing *in vivo* evidence remains limited, heterogeneous in experimental design, and insufficient to support general conclusions on safety or efficacy. Systematic metabolic analysis, long-term biosafety verification, and mechanistic exploration are still needed to facilitate the industrial translation of nanoemulsions. It is also worth noting that the broader literature suffers from heterogeneous formulations, inconsistent nanoemulsion definitions, a predominance of in vitro evidence, and a lack of standardized characterization and safety protocols, which collectively limit cross-study comparability and generalizability. Future research should focus on developing energy-saving, low-cost and clean-label nanoemulsion fabrication technologies based on natural emulsifiers. In addition, integrated *in vitro–in vivo* evaluation systems need to be established to clarify the absorption, distribution and functional mechanisms of encapsulated bioactives. Moreover, continuous technological and systematic optimization will further facilitate the large-scale application of nanoemulsions in functional food development and nutrient-delivery applications.

## Author contribution

**Zhi-Hong Zhang**: Investigation, Conceptualization, Writing – review & editing, Software, Project administration. **Kaiwei Zhang**: Investigation, Conceptualization, Writing – original draft, Software. **Xiaolan Li**: Investigation, Methodology. **Yuyue Qin**: Investigation, Methodology. **Jingkun Yan**: Investigation, Conceptualization. **Xianli Gao**: Conceptualization, Supervision, Project administration, Writing – review & editing.

## Data availability

Data will be made available on request.

## Ethical approval

In this review article, no living organisms were used to collect or obtain experimental data.

## Funding

The authors are grateful for the support provided by the 10.13039/501100001809National Natural Science Foundation of China (32572499, 32372254).

## Declaration of competing interest

The authors declare that they have no known competing financial interests or personal relationships that could have appeared to influence the work reported in this paper.
